# A Cost-Effective Approach to Creating Large Silicone Rubber Molds Using Advanced Rigid Polyurethane Foam

**DOI:** 10.3390/polym16152210

**Published:** 2024-08-02

**Authors:** Chil-Chyuan Kuo, Yi-Qing Lu, Song-Hua Huang, Armaan Farooqui

**Affiliations:** 1 Department of Mechanical Engineering, Ming Chi University of Technology, No. 84, Gungjuan Road, New Taipei City 24301, Taiwan; 2 Research Center for Intelligent Medical Devices, Ming Chi University of Technology, No. 84, Gungjuan Road, New Taipei City 24301, Taiwan; 3 Department of Mechanical Engineering, Chang Gung University, No. 259, Wenhua 1st Road, Guishan District, Taoyuan City 33302, Taiwan; 4 Center for Reliability Engineering, Ming Chi University of Technology, No. 84, Gungjuan Road, Taishan District, New Taipei City 24301, Taiwan; 5 Li-Yin Technology Co., Ltd., New Taipei City 241, Taiwan; 6 Department of Mechanical Engineering, Chhattisgarh Swami Vivekanand Technical University, Bhilai 491107, Chhattisgarh, India

**Keywords:** polyurethane foam components, silicone rubber mold, rigid, warpage, surface hardness, compressive strength

## Abstract

In practical applications, polyurethane (PU) foam must be rigid to meet the demands of various industries and provide comfort and protection in everyday life. PU foam components are extensively used in structural foam, thermal insulation, decorative panels, packaging, imitation wood, and floral foam, as well as in models and prototypes. Conventional technology for producing PU foam parts often leads to defects such as deformation, short shots, entrapped air, warpage, flash, micro-bubbles, weld lines, and voids. Therefore, the development of rigid PU foam parts has become a crucial research focus in the industry. This study proposes an innovative manufacturing process for producing rigid PU foam parts using silicone rubber molds (SRMs). The deformation of the silicone rubber mold can be predicted based on its wall thickness, following a trend equation with a correlation coefficient of 0.9951. The volume of the PU foam part can also be predicted by the weight of the PU foaming agent, as indicated by a trend equation with a correlation coefficient of 0.9824. The optimal weight ratio of the foaming agent to water, yielding the highest surface hardness, was found to be 5:1. The surface hardness of the PU foam part can also be predicted based on the weight of the water used, according to a proposed prediction equation with a correlation coefficient of 0.7517. The average surface hardness of the fabricated PU foam part has a Shore O hardness value of approximately 75. Foam parts made with 1.5 g of water added to 15 g of a foaming agent have the fewest internal pores, resulting in the densest interior. PU foam parts exhibit excellent mechanical properties when 3 g of water is added to the PU foaming agent, as evidenced by their surface hardness and compressive strength. Using rigid PU foam parts as a backing material in the proposed method can reduce rapid tool production costs by about 62%. Finally, an innovative manufacturing process for creating large SRMs using rigid PU foam parts as backing material is demonstrated.

## 1. Introduction

To streamline product development and minimize both time and cost, additive manufacturing (AM) was introduced [1], potentially transforming the manufacturing process significantly. Despite its advantages, prototypes often need to meet the specific material requirements of the final product. In response, rapid tooling (RT) technologies were developed, leveraging AM techniques to create mold inserts [2]. Recognized for its broader impact beyond just component testing, RT stands out as a crucial method for reducing costs and expediting time to market in new product development. RT, well recognized for its ability to replace traditional steel tooling, contributes to cost and time savings in the manufacturing process [3]. Currently, the industry offers various RT technologies, categorized into direct and indirect tooling [4]. Direct tooling involves crafting mold inserts directly from an AM machine, such as through selective laser sintering [5,6]. Indirect tooling involves creating mold inserts using a master pattern fabricated through various AM technologies. Soft tooling, ideal for low-volume production, utilizes materials with low hardness levels, such as silicone rubber [7] and epoxy resin composites [8]. By contrast, hard tooling is employed for higher production volumes and employs materials with higher hardness levels. Soft tooling, which uses epoxy composites with aluminum particles, silicone rubber, or low-melting-point alloys, is easier to handle than traditional mold steel tooling. Indirect soft tooling is preferred over direct tooling in new product development due to its speed, simplicity, and cost-effectiveness [9]. Silicone rubber molds (SRMs), known for their flexible and elastic characteristics, are frequently employed in this method, facilitating the fabrication of parts with intricate geometries [10]. Notably, SRMs are versatile and can be utilized for producing low-melting-point metal parts, wax patterns, and plastic parts [11].

**Figure 1:**
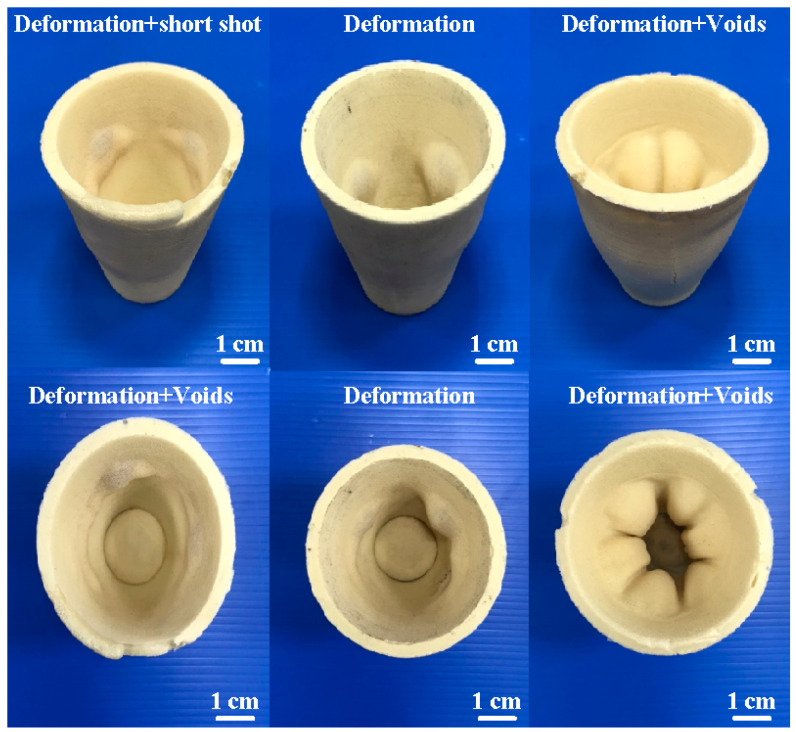
Some defects that appear in PU foam parts.

It is well known that rigid polyurethane (PU) foam parts [12] can be employed in structural foam, thermal insulation applications [13], decorative panels, packaging, imitation wood, and floral foam, as well as in models and prototypes [14]. Li et al. [15] used a self-built gas explosion testing platform to explore the quenching effect of flame-retardant polyurethane foam on a gas explosion. The results showed that polyurethane foam has an excellent flame-quenching performance. The best suppression effect can be obtained when the polyurethane foam is 20 cm long, filling 1.8 m. Zemła et al. [16] developed rigid polyurethane-foamed parts for a flame retardant. The results showed that adding 1.0 wt.% phosphorus will cause the modified rigid polyurethane foam to become a self-extinguishing material. Sture et al. [17] developed rigid polyurethane foam parts using a fourth-generation blowing agent and reactive catalysts. The results showed that the developed polyurethane-foamed parts exposed to UV light at about 1000 h resulted in no significant destruction of cellular structure deeper in the material. Ran et al. [18] investigated the grouting mechanisms of polyurethane composite materials. It was found that the geopolymer filled with the foam pores of polyurethane forms a stable consolidated body. In addition, the rigid polyurethane-foamed parts can withstand loading, cutting, or machining. Thus, the rigid polyurethane-foamed parts can be the backing materials for a sizeable rapid tool. However, conventional technology cannot produce PU foam parts with high rigidity. Some defects occurring in PU foam parts produced using conventional technology are shown in Figure 1. These defects include deformation, short shots, trapped air, warpage, flash, micro-bubbles, weld lines, and voids [19,20,21,22,23].

The traditional method for making large SRMs involves using only new silicone rubber materials [24]. This approach has two main drawbacks: it is costly, and it makes it difficult to remove the molded product from the SRMs. To address this issue, this study developed high-hardness PU foam parts to be used as backing materials for SRMs. By using these high-hardness PU foam parts, large SRMs can be produced more cost-effectively, and the molded products are easier to de-mold. This study proposed technology for developing rigid polyurethane-foamed parts using an SRM. A cost-effective method to fabricate large SRMs was demonstrated using advanced rigid PU foam parts.

## 2. Experimental Details

**Figure 2:**
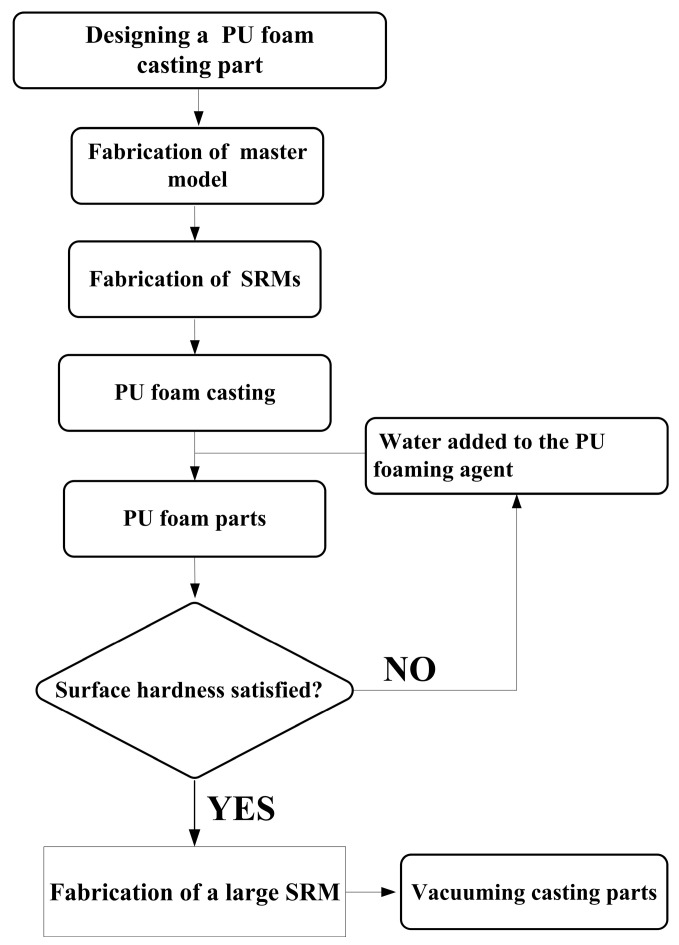
Flowchart of the experimental methodology.

**Figure 3:**
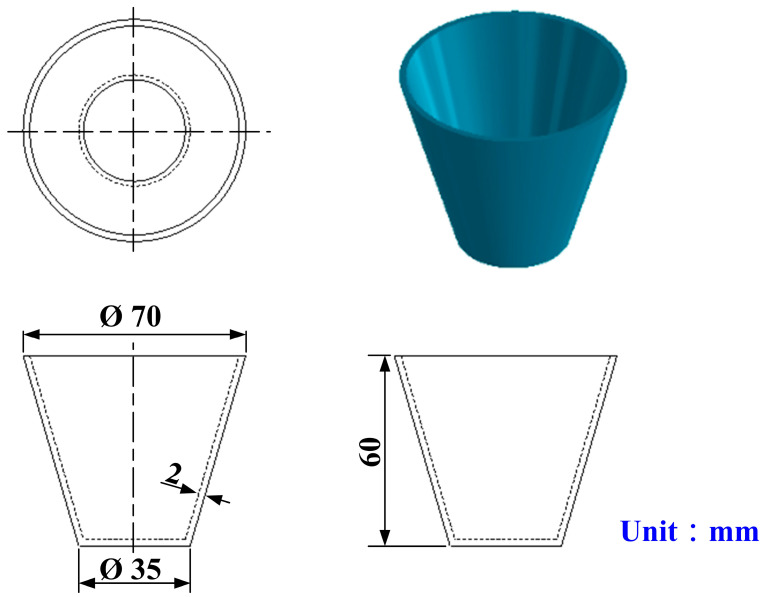
Three-dimensional CAD model and dimensions of PU foam parts.

Figure 2 shows the flowchart of the experimental methodology. The research items include the designing of a PU foam casting part, fabrication of master model, fabrication of SRMs, water added to the PU foaming agent to develop rigid polyurethane-foamed parts, evaluation of the surface hardness satisfied of the fabricated polyurethane-foamed parts, and, finally, fabrication of a large SRM using fabricated rigid polyurethane-foamed parts as backing materials. Figure 3 shows the 3D CAD model and dimensions of PU foam parts. In this study, the PU foam part is a water cup. The water cup was chosen as the test specimen, mainly because the core and cavity mold inserts of the water cup are obvious. The wall thickness, top diameter, bottom diameter, and height are 2 mm, 70 mm, 35 mm, and 60 mm, respectively. In this study, a fused deposition modeling machine (Mark Two, Markforged Inc., Waltham, MA, USA) was employed to print the master model using carbon fiber composite filament stocks (Onyx, Markforged Inc., Waltham, MA, USA). The main reason is that the master models produced have excellent surface quality and mechanical properties.

Silicone rubber (KE-1310ST, Shin Etsu Inc., New Taipei City, Taiwan) and a curing agent (CAT-1310S, Shin Etsu Inc., New Taipei City, Taiwan) were mixed in a weight ratio of 10:1 to manufacture an SRM. The mixing process was conducted using a vacuum machine (F-600, Feiling, Inc., New Taipei City, Taiwan) to eliminate air bubbles. The vacuum process used to eliminate air bubbles involved setting the vacuum pressure to −80 kPa and applying it for 5–10 min immediately after mixing the mixtures. This timing captured air bubbles introduced during mixing process. These parameters were optimized through preliminary experiments to ensure effective bubble removal.

PU 51 foam was (Puff 101 Dino Inc., New Taipei City, Taiwan) used to cast parts in the SRM. PU 51 foam, used to cast parts in the SRM, has a density of 0.64 g/cm^3^, a Shore hardness of 55 D, a tensile strength of 44 MPa, an elongation at break of 18%, a flexural strength of 75 MPa, and a compression strength of 84 MPa.

**Figure 4:**
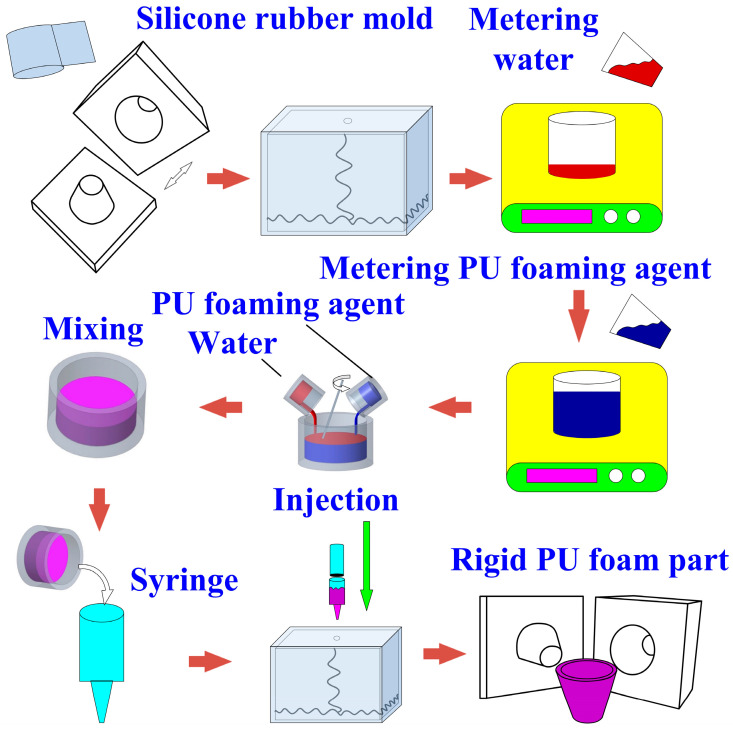
An innovative manufacturing process for manufacturing rigid PU foam parts through an SRM.

Figure 4 shows the innovative manufacturing process for manufacturing rigid PU foam parts through an SRM. First, tape was used to ensure the SRM did not expand due to the foaming agent. Next, water and PU foam with a weight ratio of 5:1 were weighted, combined, and mixed for less than two minutes. Subsequently, the mixture was poured into an injection syringe (Hamilton Inc., Reno, NV, USA) and injected into the cavity of the SRM. After about 1.5 h, the foamed parts were removed from the mold, and the excess water was allowed to evaporate. These steps produced rigid PU foam parts.

**Figure 5:**
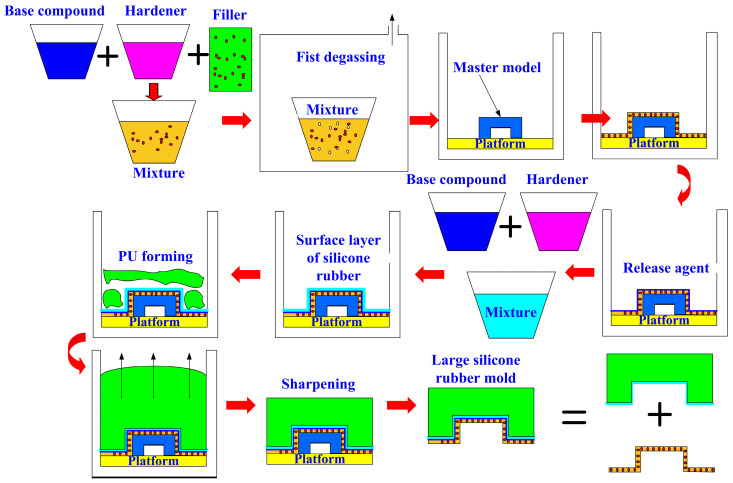
Manufacturing process diagram of a large silicone rubber mold using a rigid polyurethane-foamed part as a backing material.

Figure 5 shows the manufacturing process diagram of a large silicone rubber mold using a rigid polyurethane-foamed part as a backing material. The manufacturing process includes fabrication of silicone rubber mold, applying the recipe proposed to create backing material, and sharpening the PU-foamed backing material. The base compound (XK-019402N POLYOL, Axson Technologies Inc., New Taipei City, Taiwan) and hardener (XK-019402 ISOCYANATE, Axson Technologies Inc., New Taipei City, Taiwan) were mixed in a weight ratio of 1:2. Finally, the liquid acrylonitrile butadiene styrene (ABS) resin was employed as the vacuum casting to validate the large fabricated SRM.

**Figure 6:**
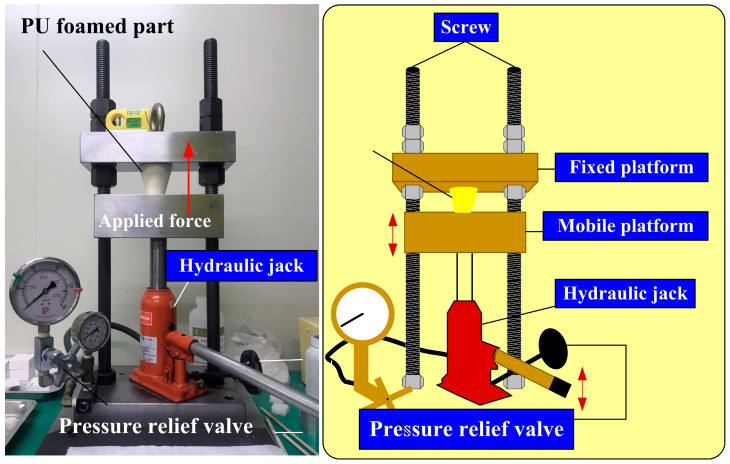
Experimental setup for compression tests of PU foam parts.

**Figure 7:**
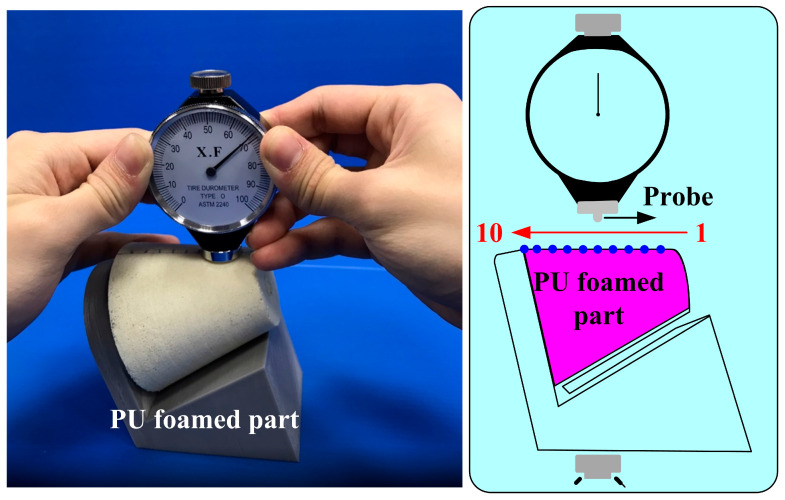
Experimental setup for surface hardness tests of PU foam parts.

A digital height gauge (Mitutoyo Inc., Shimogurimachi, Japan) was used to measure the deformation of SRMs. In general, the ASTM D2240 [25] is a standard for measuring the hardness of materials such as rubber, elastomers, or plastics. In addition, the three-point bending (RH-30, Shimadzu Inc., Kyoto, Japan) test and Shore O surface hardness test (MET-HG-A, SEAT Inc., New Taipei City, Taiwan) were performed to assess the weld quality. To evaluate the quality of the fabricated rigid polyurethane-foamed parts, both compression and surface hardness tests were performed. Figure 6 shows the experimental setup for compression tests of PU foam parts. The applied pressure for the PU foam parts is approximately 689 kPa. Figure 7 shows the experimental setup for surface hardness tests of PU foam parts. There are three measurement lines on the outer surface of PU foam parts. Each measurement line is 120° apart, and each measurement line has ten measurement points.

## 3. Results and Discussion

**Figure 8:**
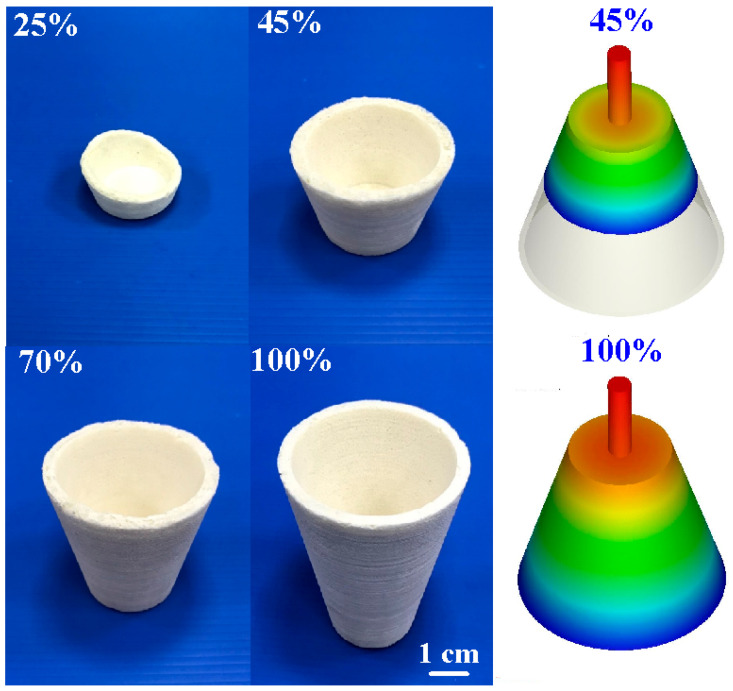
Short-shot experimental results of PU-foamed parts using a silicone rubber mold.

Figure 8 shows the short-shot experimental results of PU foam parts using a silicone rubber mold. The filling process exhibited smooth flow. A complete PU foam part was formed with 15 g of PU foaming agent because defects, such as weld lines [26] or meld lines [27] were not presented in the filled parts.

**Figure 9:**
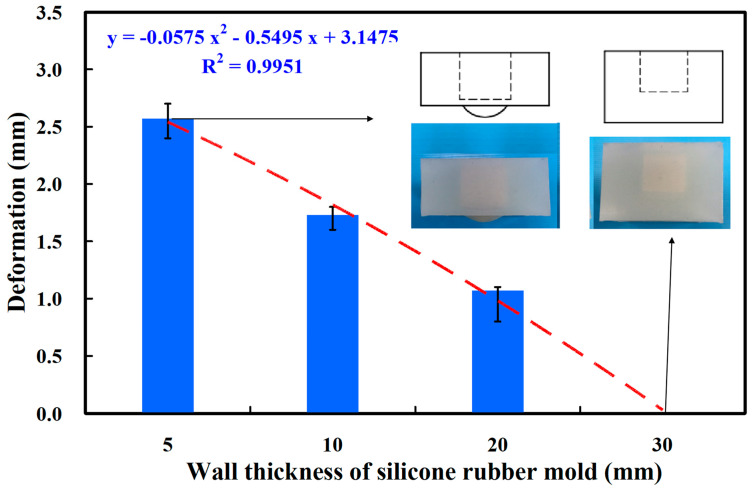
The relationship between the deformation of silicone rubber mold and the wall thickness of silicone rubber mold.

**Figure 10:**
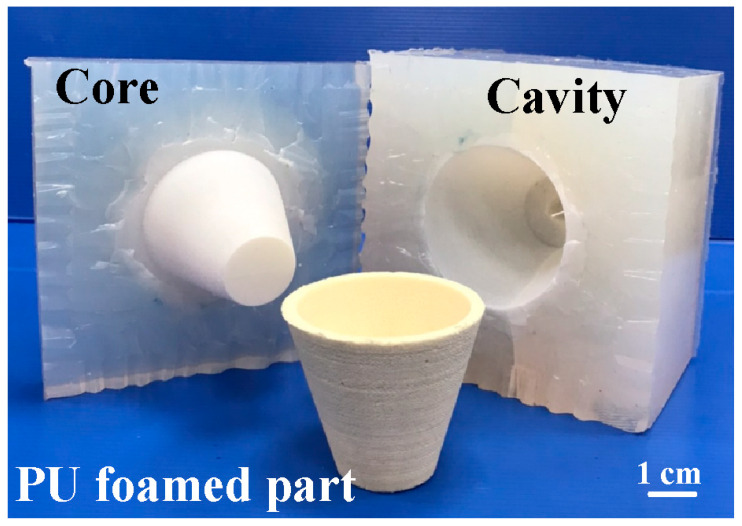
The silicone rubber mold for forming PU parts.

**Figure 11:**
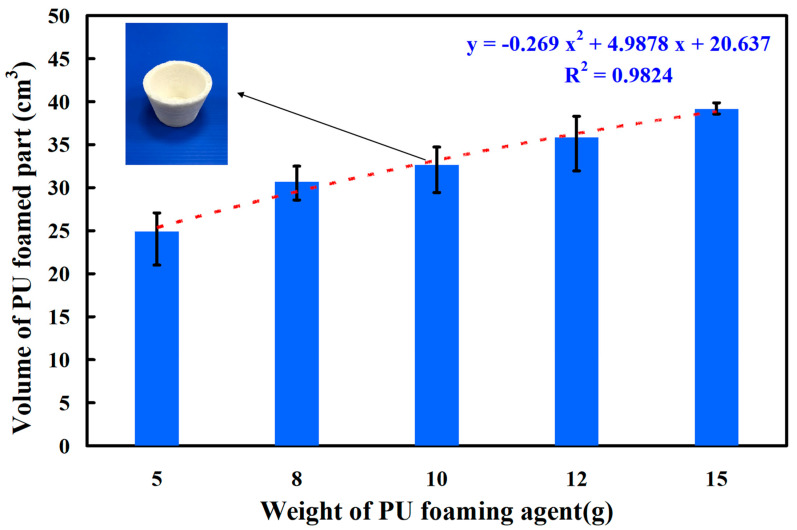
The relationship between the volume of PU foam part and the weight of PU foaming agent.

Curve fitting is the process of finding a mathematical function that best matches a set of data points by adjusting model parameters to minimize the difference between the model and the data. Silicone rubber molds were employed because a release agent is not required during the PU foaming process [28,29]. Deformation of the silicone rubber mold, however, occurred during the PU foaming process. Figure 9 presents the relationship between the deformation of silicone rubber mold and the wall thickness of silicone rubber mold. The deformation of silicone rubber mold (y) correlated with the wall thickness of silicone rubber mold (x) as y = −0.0575 x^2^ − 0.5495 x + 3.1475; the correlation coefficient was 0.9951 [30]. This equation is based on the curve fitting method. The deformations of the SRM were approximately 2.57 mm, 1.73 mm, 1.07 mm, and 0 mm when the silicone mold wall thicknesses were 5 mm, 10 mm, 20 mm, and 30 mm, respectively. To prevent deformation of the silicone mold during the PU foaming process, the silicone mold needed to have walls that were at least 30 mm thick. Figure 10 shows the silicone rubber mold for forming PU parts. The wall thickness of forming PU parts is 2 mm. Figure 11 shows the relationship between the forming volume of PU foam part and the weight of the PU foaming agent. The results showed that the volume of PU foam parts is approximately 24.87 cm^3^, 30.66 cm^3^, 32.64 cm^3^, 35.86 cm^3^, and 39.14 cm^3^ when the weight of PU foaming agent is 5 g, 8 g, 10 g, 12 g, and 15 g. As can be seen, the volume of PU foam part (y) can be determined by the weight of the PU foaming agent (x) according to the trend equation of y = −0.269 x^2^ + 4.9878 x + 20.637 with an correlation coefficient of 0.9824.

**Figure 12:**
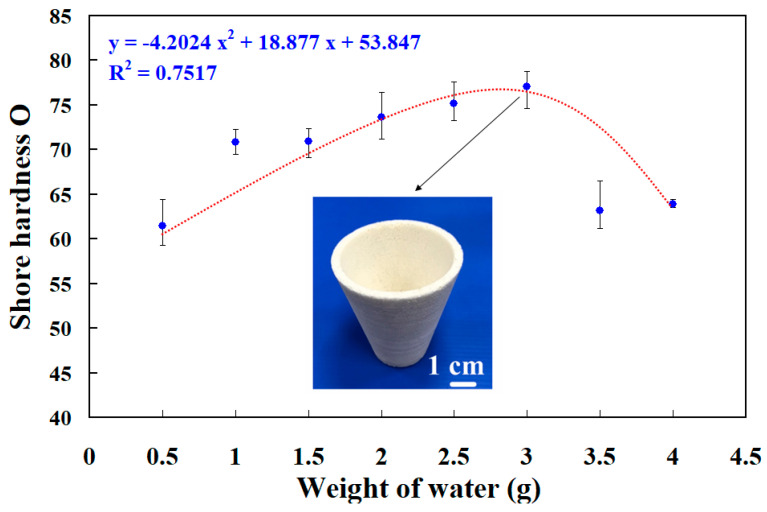
The relationship between the surface hardness and weight of this added water.

**Figure 13:**
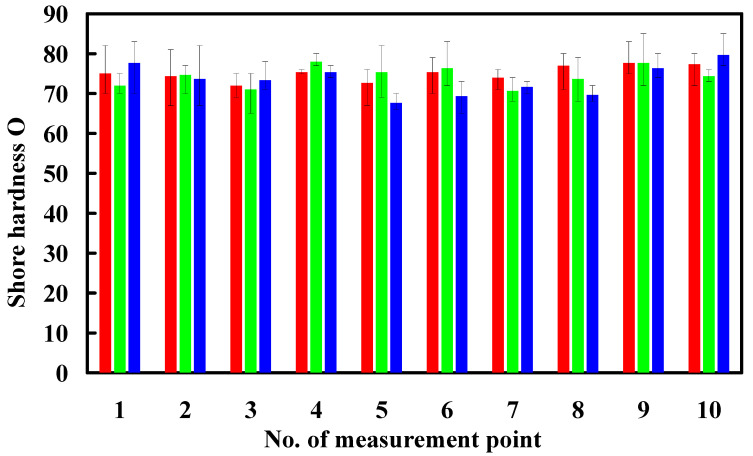
Surface hardness of a PU foam part with a weight ratio of foaming agent to water of 5:1.

A foaming agent weight of 15 g was used in this study. To understand the effect of adding water inside the PU foaming agent on the surface hardness of the PU foam produced in this process, 0.5 g, 1 g, 1.5 g, 2 g, 2.5 g, 3 g, 3.5 g, and 4 g of water were used with the PU foaming agent. Figure 12 presents the relationship between the surface hardness and weight of this added water. The average surface hardness of the PU foam parts increased when 0.5 g to 3 g of water were added inside the PU foaming agent. However, the average surface hardness of the molded PU foam parts decreased when the weight of water added inside the PU foaming agent exceeded 3 g. The molded PU foam parts had the highest average surface hardness when the weight of water added inside the PU foaming agent was 3 g. Therefore, the weight ratio of foaming agent to water of 5:1 produced the highest surface hardness. In addition, the surface hardness of the PU foam part (y) could be determined by the water weight (x) according to the prediction equation of y = −4.2024 x^2^ + 18.877 x + 53.847 with a correlation coefficient of 0.7517. Figure 13 shows the surface hardness of a PU foam part with a weight ratio of foaming agent to water of 5:1. The average surface hardness of the surface of this PU foam part was about Shore O 75.

**Figure 14:**
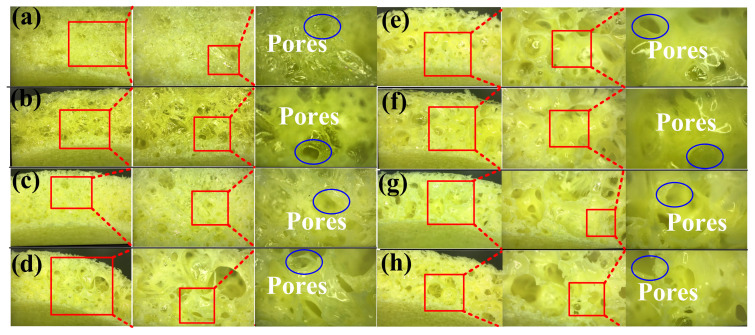
Cross-sectional structure of foam parts made by adding (**a**) 0.5 g, (**b**) 1g, (**c**) 1.5 g, (**d**) 2 g, (**e**) 2.5 g, (**f**) 3 g, (**g**) 3.5 g, and (**h**) 4 g of water to 15 g foaming agent.

Figure 14 shows the cross-sectional structure of foam parts made by adding 0.5 g, 1g, 1.5 g, 2 g, 2.5 g, 3 g, 3.5 g, and 4 g of water to the 15 g foaming agent. The results showed that the cross-sectional structure of the foam parts made with 1.5 g of water added to 15 g of foaming agent has the least internal pores because the interior of the foamed part made is the densest. The findings reveal that adding 1.5 g of water to 15 g of foaming agent results in foam parts with the least internal pores, indicating a denser interior structure. This phenomenon occurs because water plays a crucial role in nucleating gas bubbles during the foaming process; precise control over water content influences pore formation, affecting the overall density and structural integrity of the foam parts.

**Figure 15:**
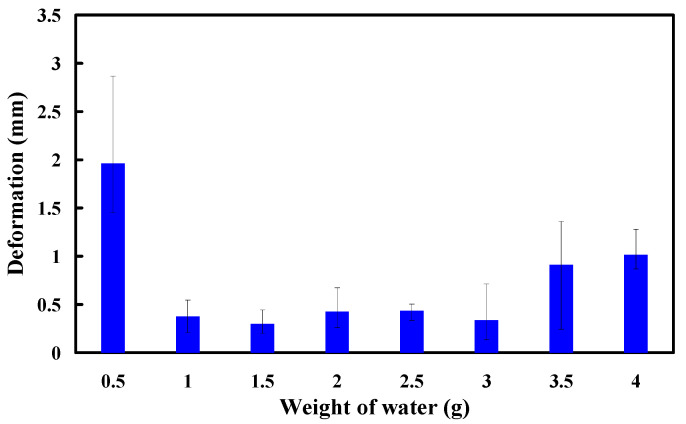
The relationship between the deformation and weight of water.

To understand the effect of adding water inside the PU foaming agent on the compressive strength of PU foam parts during the PU foaming process, this study added 0.5 g, 1 g, 1.5 g, 2 g, 2.5 g, 3 g, 3.5 g, and 4 g of water in the PU foaming agent to make PU foam parts. Figure 15 shows the relationship between the deformation and weight of water. The results showed that the deformation of the PU foam parts is approximately 1.96 mm, 0.38 mm, 0.3 mm, 0.42 mm, 0.43 mm, 0.34 mm, 0.9 mm, and 1.02 mm when 0.5 g, 1 g, 1.5 g, 2 g, 2.5 g, 3 g, 3.5 g, and 4 g of water are added to the PU foaming agent. This study found that the deformation amounts of the PU foam parts were very close when 1.5 g and 3 g of water were added to the PU foaming agent. Considering the surface hardness and compressive strength of the PU foam parts, it was found that the PU foam part had excellent mechanical properties when the PU foaming agent was added to the 3 g of water. Therefore, 3 g of water was used to add inside the PU foaming agent to make the back material of a large silicone rubber mold. The developed rigid PU foam parts may have competitive flame-retardant properties compared to other common materials used in similar applications. By incorporating additives like phosphorus-based compounds, or mineral fillers, PU foams can achieve effective flame resistance, low smoke generation, and self-extinguishing capabilities. These properties make PU foam a versatile choice for applications where fire safety standards are crucial, offering a balance of performance, thermal insulation, and formability.

**Figure 16:**
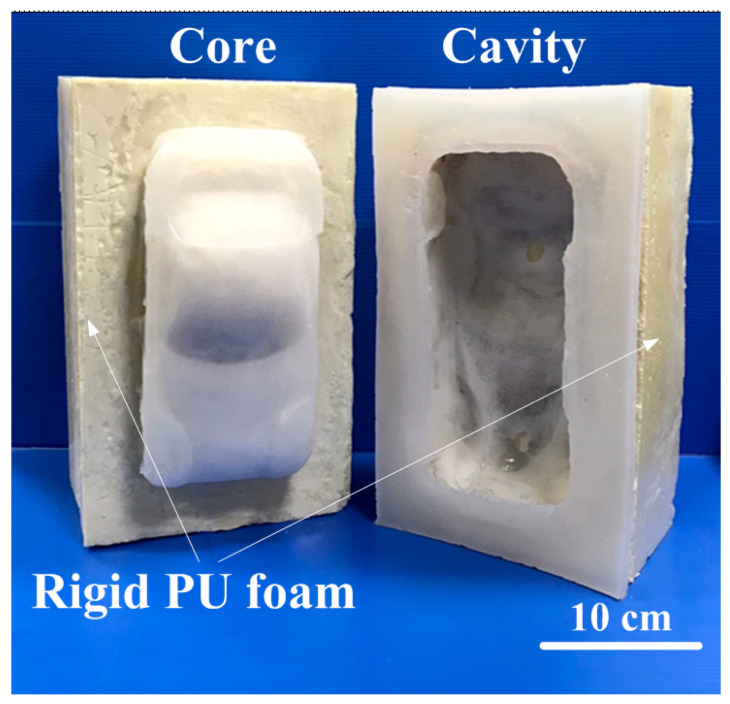
A cost-effective large rapid tool developed in this study and a molded part.

The traditional method for making large SRMs uses only new silicone rubber materials, which are costly and make it difficult to remove the molded product. To solve this, the study developed high-hardness PU foam parts as backing materials for SRMs. Using these PU foam parts makes large SRMs cheaper to produce and easier to de-mold. This study proposed technology for developing rigid polyurethane-foamed parts with SRMs, offering a cost-effective method for fabricating large SRMs. Figure 16 shows a cost-effective large rapid tool developed in this study and a molded part. The SRM plays a pivotal role in prototyping, enabling the iteration of designs for testing and enhancement. Their versatility optimizes manufacturing, ensuring the swift production of superior-quality components and prototypes. With silicone rubber molds at the helm, industries enjoy streamlined processes, marked by consistency and exactitude in design replication through vacuum casting technology.

**Figure 17:**
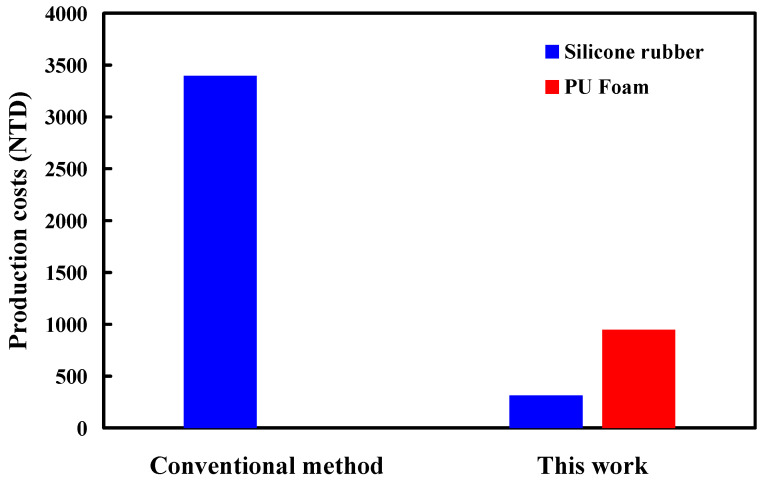
Comparison of production costs for a large rapid tool.

Figure 17 shows a comparison of production costs for a large rapid tool. Taking the car shell of a sports car as an example, the length, width, and height of the sports car shell are 235 mm, 113 mm, and 64 mm. The thickness of the sports car is approximately 2.5 mm. The total production cost of the entire set of molds is approximately NTD 3396 using traditional methods to produce large rapid molds. However, the total production cost of the entire set of molds is only approximately NTD 1261 by the method proposed in this study. The results showed that the proposed method can save about 62% of the total mold production cost. It should be noted that this large rapid tooling [31,32,33,34] was fabricated with rigid polyurethane-foamed parts as backing material. Thus, this method has application potential in the industry because of the reduction in the production cost increases with increasing the size of the rapid tool. Rigid PU foam parts attain notable levels of thermal and acoustic insulation even with minimal thickness. Available in sheets, blocks, and molded pieces, rigid polyurethane foam can be tailored to client specifications regarding form, texture, color, and more. Its versatility extends to applications such as insulation in construction, industrial refrigeration, pipes, tanks, heaters, etc. Additionally, it finds utility in structural foam, imitation wood, decorative panels, packaging, floral foam, and even in the creation of models and prototypes. Based on comprehensive experiments, the outcomes of this study suggested potential applications in the research and development phase. Broadly, the use of sprayed rigid PU foam components finds diverse applications in construction and in various industries. Additionally, employing PU shell molding proves cost-effective for large volume production. The utilization of rigid PU foam elements as backing materials facilitates the creation of a cost-effective large rapid tool for vacuum casting. Further processing of vacuum-cast parts, including assembly, polishing [35], grinding [36], drilling [37], cutting [38], or tapping [39], is under current investigation. Further research could also focus on optimizing processing techniques for rigid PU foam parts, such as refining the spraying process for enhanced uniformity and efficiency to improve production yields and reduce costs. Investigating additional industries or niche applications where rigid PU foam could offer benefits, such as in the aerospace, automotive, marine, or medical sectors. This could involve conducting feasibility studies, material testing, and prototype development to assess the suitability and performance of rigid PU foam in these contexts. In addition, it is necessary to conduct an investigation of the environmental impact [40,41] of rigid PU foam production and disposal and explore ways to improve sustainability throughout the product lifecycle. This could include research into alternative raw materials, recycling or upcycling strategies, and the development of eco-friendly manufacturing processes.

Green manufacturing refers to the production of goods with minimal environmental impact by integrating practices like resource efficiency, renewable energy use, and recycling [42,43,44,45,46,47]. It emphasizes the entire product lifecycle, from design to disposal, promoting sustainability and reducing the carbon footprint. By adopting these principles, companies can align economic growth with environmental responsibility, contributing to a healthier planet for future generations. Based on the research outcomes, this study aligns with green manufacturing principles and demonstrates significant potential applications in the industry. The findings of this research support Sustainable Development Goals (SDGs) 7 (Affordable and Clean Energy), 9 (Industry, Innovation, and Infrastructure), 10 (Reduced Inequality), 11 (Sustainable Cities and Communities), and 12 (Responsible Consumption and Production) [48,49,50]. Green smart manufacturing merges green manufacturing practices with cutting-edge technologies like artificial intelligence and the Internet of Things to optimize resource usage and enhance productivity while minimizing environmental impact [51]. By leveraging data-driven approaches and automation, it aims to create efficient and sustainable manufacturing processes. Ultimately, green smart manufacturing strives to achieve both environmental sustainability and economic efficiency in the manufacturing industry. Ongoing investigations into these matters will be presented in a subsequent study.

## 4. Conclusions

PU foam parts refer to components made from PU foam, a versatile and lightweight material formed by combining two liquid components that react chemically to produce foam. This foam exhibits properties such as flexibility, resilience, and insulation capabilities. PU foam parts are used in a wide range of industries, including construction, insulation, furniture, packaging, decorative panels, floral arrangements, and model and prototype creation. The adaptable nature of PU foam allows it to be molded into various shapes and sizes to meet specific needs. The main conclusions from the experimental work in this study are as follows:

1. The deformation of SRM (y) can be determined by the wall thickness of silicone rubber mold (x) according to the trend equation of y = −0.0575 x^2^ − 0.5495 x + 3.1475 with a correlation coefficient of 0.9951. In addition, the volume of a PU foam part (y) can be determined by the weight of a PU foaming agent (x) according to the trend equation of y = −0.269 x^2^ + 4.9878 x + 20.637 with a correlation coefficient of 0.9824.
2. The weight ratio of foaming agent and water with the highest surface hardness can be achieved at 5:1. The surface hardness of the PU foam part (y) can be determined by the water weight (x) according to the prediction equation of y = −4.2024 x^2^ + 18.877 x + 53.847 with a correlation coefficient of 0.7517. The average surface hardness of the surface of the fabricated PU foam part has a Shore O hardness value of about 75.
3. The foam parts made with 1.5 g of water added to 15 g of foaming agent have the least internal pores because the interior of the foamed part made is the densest. The PU foam part had excellent mechanical properties when the PU foaming agent was added to 3 g of water based on both the surface hardness and compressive strength of the PU foam parts. Taking the car shell of a sports car as an example, the proposed method using rigid polyurethane-foamed parts as backing materials can save about 62% of the rapid tool production cost.

## Data Availability

Data are contained within the article.
